# Center of Pressure Displacement of Standing Posture during Rapid Movements Is Reorganised Due to Experimental Lower Extremity Muscle Pain

**DOI:** 10.1371/journal.pone.0144933

**Published:** 2015-12-17

**Authors:** Shinichiro Shiozawa, Rogerio Pessoto Hirata, Thomas Graven-Nielsen

**Affiliations:** Laboratory for Musculoskeletal Pain and Motor Control, Center for Sensory-Motor Interaction (SMI), Department of Health Science and Technology, Faculty of Medicine, Aalborg University, Aalborg, Denmark; Universite de Nantes, FRANCE

## Abstract

**Background:**

Postural control during rapid movements may be impaired due to musculoskeletal pain. The purpose of this study was to investigate the effect of experimental knee-related muscle pain on the center of pressure (CoP) displacement in a reaction time task condition.

**Methods:**

Nine healthy males performed two reaction time tasks (dominant side shoulder flexion and bilateral heel lift) before, during, and after experimental pain induced in the dominant side vastus medialis or the tibialis anterior muscles by hypertonic saline injections. The CoP displacement was extracted from the ipsilateral and contralateral side by two force plates and the net CoP displacement was calculated.

**Results:**

Compared with non-painful sessions, tibialis anterior muscle pain during the peak and peak-to-peak displacement for the CoP during anticipatory postural adjustments (APAs) of the shoulder task reduced the peak-to-peak displacement of the net CoP in the medial-lateral direction (P<0.05). Tibialis anterior and vastus medialis muscle pain during shoulder flexion task reduced the anterior-posterior peak-to-peak displacement in the ipsilateral side (P<0.05).

**Conclusions:**

The central nervous system in healthy individuals was sufficiently robust in maintaining the APA characteristics during pain, although the displacement of net and ipsilateral CoP in the medial-lateral and anterior-posterior directions during unilateral fast shoulder movement was altered.

## Introduction

The link between knee pain and motor control is clinically relevant since pain around the knee joint affects body control in adolescent [1], young adults [2] and elderly people [3] which increases the risk of falls in elderly [4]. Previous studies have shown that knee pain affects the neuromuscular function of knee related muscles by reducing the muscle activity of thigh muscles during gait [5], stair climbing [6], and forward lunge [7]. Moreover, knee pain affects the spinal reflex and the motor neuronal firing rate of the m. quadriceps femoris [8,9].

Compared with asymptomatic subjects, knee osteoarthritis (OA) patients showed reduced knee flexion moment during gait [10,11]. During gait and forward lunge, the peak moment and peak angle of the knee joint were reduced due to experimental knee pain when compared with pain free conditions [5,7]. Experimental pain close to the knee joint also reduces maximum voluntary contraction force in both isometric and isokinetic contractions [12,13] and hampers the force steadiness during isometric contractions [14]. Moreover, experimental knee-related pain increased the velocity of the center of pressure (CoP) displacement in the anterior-posterior and medial-lateral directions during quiet standing when compared with non-painful standing conditions [15]. This illustrates that pain close to the knee joint not only induces alteration in the neuromuscular function, but also affects the final motor output observed in both CoP displacement and velocity.

Anticipatory postural adjustments (APAs), defined as the time window from -50 to +150ms from the prime mover onset, are generated by the central nervous system in a feed-forward manner to stabilise the body’s center of gravity [16,17]. Differences in performance during reaction time tasks are associated with the magnitudes of anticipatory postural adjustments [18], especially in muscles of the frontal part of the body [19]. Furthermore, there is a correlation between APAs and motor performance during reaching tasks [20]. Longer APAs were associated with balance impairments in elderly [21] and, poor body stabilization during such reaction time tasks was associated with impaired APAs responses in children with development coordination disorder [22]. Center of pressure parameters, such as displacement and velocity, have been widely used to quantify reorganised postural control strategies during knee pain conditions [23]. For instance, lateral sway of CoP during static standing in knee OA patients was greater than control subjects [23]. In a step down task, the time for CoP counter response during APA was increased in knee OA patients compared with control subjects [24]. Moreover, the displacement of CoP during single leg standing was smaller in knee OA patients with stronger vs weak m. quadriceps femoris [25]. Severe knee OA patients have also impaired postural stability when environmental conditions are adverse (such as unstable surface and impaired visual information) compared with less severe patients [3]. Although it seems clear these patients have balance impairments, the source of such problem cannot be easily isolated. For example, structural changes at the knee and/or pain could per se affect balance. From a clinical perspective it is important to know if the impaired balance can be improved by reducing the pain. However, current clinical study designs cannot extract the contribution of each of these parameters on the impairments observed. Experimental pain models have been extensively used in healthy subject to understand only the effects of pain in an otherwise healthy system [26]. Although the validity of these methods in clinical settings can be discussed, experimental pain models have mimic several chronic pain conditions [27–30]. Previous studies showed that experimental knee-related pain alters differently the muscle onset and activation levels during reaction tasks depending on the task (unilateral upper extremity vs bilateral lower extremity) [31,32]. However, it was not clear if such changes actually improve balance stability (CoP parameters) and how much each limb contributes to this possible improvement. Specifically in balance, it is not fully understood how knee-related muscle pain per se, without any other confounding factors, affects CoP parameters during APA reposes to reaction time tasks using lower or upper limbs as prime movers. Such an approach could provide a better understanding of the mechanism underlying the sensorimotor interactions of balance during pain.

The aim of this study was to investigate how experimental pain close to the knee joint affects APA responses in the CoP parameters. It was hypothesized that knee-related muscle pain will 1) increase the CoP displacement and velocity on the painful side, and 2) decrease the CoP displacement and velocity on non-painful side to compensate the increased CoP parameters from the painful side and, therefore, maintain balance stability.

## Materials and Methods

### Subjects

In this study nine healthy males were included without known musculoskeletal disorders (mean ± standard deviation, 29 ± 5 years [range 23–38 years], 179 ± 9 cm tall, and 77 ± 12 kg body mass). Given the gender variability in motor responses to pain, the present study only evaluated healthy male subjects, which restrict the generalization of the results to this population. Participants signed an informed consent prior participating in the study. The study was performed according to the Declaration of Helsinki and was approved by the local ethical committee (The Ethics Committee of The North Denmark region, N-20080022).

### Experimental protocol

The subjects stood with preferred foot position on two force plates, with the arms relaxed along the side and looked at a circle (distance 4 m, diameter 5 cm) in front of them. The feet position was marked with tape to assure that the same position was used during all trials. The subjects performed, as quickly as possible, either a unilateral shoulder flexion (minimum 90 degrees flexion, dominant side) or bilateral heel lift movement after a go signal (sound cue) was presented. Before the go signal (randomly assigned with 2–6 s interval), a warning signal was delivered to the subjects to maintain a quiet standing posture before participants performed the tasks. The time of data collection from the warning signal to end of recording was 10 seconds in each trial. The participants performed 20 reaction tasks alternating between trials of unilateral shoulder joint flexion and bilateral ankle joints plantar flexion. The data collection was performed in two days with at least one week in between and consisted of 6 different sessions for each day: (i) 1^st^ baseline, (ii) non-painful session: isotonic saline injection, (iii) post-isotonic saline injection, (iv) 2^nd^ baseline, (v) painful session: hypertonic saline injection, and (vi) post-hypertonic saline injection. Injections were given in the dominant side and defined as ipsilateral in the further description. One isotonic and one hypertonic saline injection were given per day in the same muscle (either m. vastus medialis or m. tibialis anterior). The order of the painful and non-painful injections was blinded for all participants.

### Detection of target movement onset

**Fig 1:**
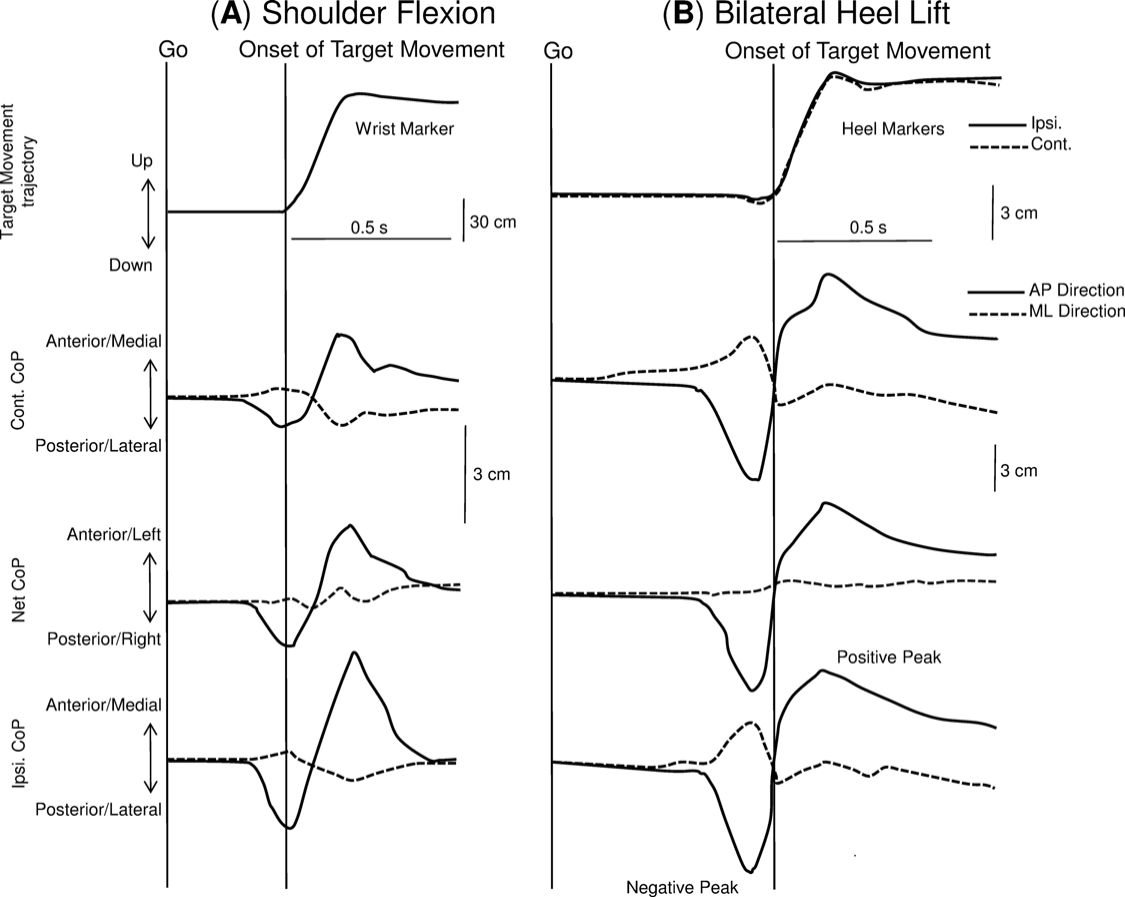
Target movement trajectory and three CoP displacements for the anterior-posterior and the medial-lateral CoP directions during the unilateral shoulder flexion task (A) and bilateral heel task (B). Onset of the target movement is illustrated as a vertical line.

Reflective markers (2 cm in diameter), tracked by eight infrared cameras (Qualysis Medical AB, Gothenburg, Sweden) each sampled with 500 Hz, were attached bilaterally on the following palpable anatomical landmarks: iliac crest, medial condyle of femur, medial malleoli of the ankle, calcanei, and the first metatarsal head. On the dominant side, markers were attached to the lateral supra condylar prominence of the elbow, acromion and styloid process of the radius. The wrist movement during the unilateral shoulder flexion task was defined by the displacement of the marker placed on the styloid process of the dominant radius, and the heel movement during the bilateral heel lift task was defined by the markers’ displacement in both calcanei. The shoulder joint angle was defined by the two segments, dominant brachium (a line by the lateral supra condylar prominence of dominant elbow marker and the dominant acromion marker), and trunk line (a line by the dominant acromion marker and the dominant iliac crest marker). The ankle joint angle was estimated by the angle between the shank (a line between the marker on the medial condyles of the femur marker and the marker on the medial malleoli) and foot (a line by the heel marker and the first metatarsal head prominence marker [31]. Peak flexion and peak angular velocity for both shoulder and ankle joint were calculated to verify if the movement pattern was maintained during all conditions. The movement onset after the ‘go signal’, defined as reaction time, for each trial was automatically identified as the time point where the relevant marker position was increased by 3 standard deviations from the quiet standing value (calculated in a 100ms window immediately after the ‘go signal’, Fig 1). Data from the first trial of unilateral shoulder flexion task and bilateral heel lift task in each session were discarded due to the variability found on those trials. Additionally, if in any trial the subjects fail to react properly to the ‘go signal’, the trial was excluded from the analysis. The movement onset was used to determine the APA time window used for the CoP analyses, which started at 50ms prior movement onset until 150ms after movement onset.

### Center of pressure recordings and data analysis

The ground reaction forces and the torques from both force platforms were amplified, low-pass filtered (10 Hz) and sampled at 2 kHz. The CoP under the ipsilateral, contralateral foot, and the net CoP were calculated [33] after the recording of the ground reaction forces and the torques from the two force plates (AMTI, USA). The CoP displacement was split into anterior-posterior (AP) and medial-lateral (ML) directions, allowing better characterisation of ankle and hip strategy during postural control [34–36]. The time points for the negative and positive peaks in the AP direction of CoP displacement were automatically estimated, and used as a time window for extraction of other CoP parameters. The first negative and positive peak value of the CoP displacement after the "go" signal were detected. The first negative peak was interpreted as the postural adjustments prior movement onset which usually occurred between -50ms to 150ms from movement onset (APA window). The first positive peak is related to the end of the movement (Fig 1) and it is usually occurring after the APA window. Understanding the relationship between both the anticipatory and focal movement components of the postural adjustments better quantifies the possible alterations in postural control due to pain. Therefore the time window between the two peaks was used for the CoP analysis. The time points for the negative and positive peaks of CoP displacement were also calculated relative to the onset of target movement and defined as relative time-to-peak for ipsilateral, contralateral and net CoP. In the bilateral heel lift task, the relative time-to-peak of the net CoP was estimated by the mean onset value from both calcaneus markers. CoP displacement between the first negative peak and the first positive peak of both AP and ML directions was calculated and defined as peak-to-peak displacement for ipsilateral, contralateral, and net CoP (Fig 1). Additionally, the peak-to-peak center of pressure velocity (average) in both directions for ipsilateral, contralateral, and net CoP was calculated by dividing the peak-to-peak displacement by its respective time interval.

### Experimental muscle pain

Muscle pain was induced in the dominant side by intramuscular injections of sterile hypertonic (1 ml, 5.8%). Injection of isotonic saline (1 ml, 0.9%) in the same side was used as control (non-painful) condition. A 2 ml plastic syringe with a disposable needle (27G, 13 mm) was used for the injections. The VM injection site was 5 cm proximal and 5 cm medial to the medial edge of the patella, and the TA injection site was 15 cm distally from the corner of patella. Previous studies applying experimental pain in the VM [31,37] and TA [31] muscles revealed that both muscles have important roles controlling posture during quiet standing and perturbations. The experimental pain intensity was updated during the interval of each trial on a 10 cm electronic visual analogue scale (VAS) where 0 cm indicated ‘no pain’ and 10 cm anchored ‘maximum pain’ and the scale could be adjusted with handheld slider. The VAS signal was recorded continuously (0.5 Hz sampling) and the average VAS score within each 10-s trial was extracted. The maximum pain for each saline injection was extracted. In the intervals between trials the subjects were asked to update the VAS.

### Statistics

All results are reported as mean ± standard error of the mean (SEM). Maximum VAS scores during the trials were assessed using a paired t-test comparing control and pain sessions. A three-way repeated measures analysis of variance (RM-ANOVA) with *side* (ipsilateral and contralateral), *painful muscle* (vastus medialis and tibialis anterior muscle), and *saline* (isotonic and hypertonic) was applied in the baseline session data to evaluate the CoP asymmetry between feet. The effect of pain was assessed with a three-way RM-ANOVA with factors: *saline*, *time* (baseline, injection, and post-injection), and *painful muscle* on the following parameters: movement reaction time (unilateral shoulder flexion and bilateral heel lift task), relative CoP negative and positive time-to-peak, AP and ML CoP displacement and the velocity. The CoP parameters for ipsilateral, contralateral, and net CoP were normalised (%) by baseline values before analysis. The Newman-Keul’s (NK) post-hoc test was applied when the main RM-ANOVA indicated significant difference. Significance was accepted at P < 0.05.

## Results

### Pain perception

The maximum VAS score after the VM injections was 0.3 ± 0.1 cm (isotonic saline) and 4.7 ± 0.8 cm (hypertonic saline). The maximum VAS score after the TA injections was 0.4 ± 0.1 cm (isotonic saline) and 4.7 ± 0.6 cm (hypertonic saline). The maximum VAS score after hypertonic saline for the both VM and TA injections demonstrated a significantly higher score than the control injections (t-test: P < 0.01).

### Target movement

The average onset of target movement from the go-signal (reaction time) is shown in Table 1. Reaction time of unilateral shoulder flexion and bilateral heel lift movement was not significantly affected by pain. While performing fast unilateral shoulder flexion, no significant differences were found in the: (i) peak angle between injections applied in the VM muscle (hypertonic 105.5 ± 19.7 degrees vs isotonic 106.9 ± 16.3 degrees) or TA muscle (hypertonic 111.5 ± 6.2 degrees vs isotonic 116.2 ± 4.4 degrees) and (ii) the peak angular velocity between type injections applied in the VM muscle (hypertonic 468.0 ± 24.3 degrees/s vs isotonic 491.3 ± 23.1 degrees/s) or TA muscle (hypertonic 494.0 ± 23.7 degrees vs isotonic 500.2 ± 22.1 degrees/s). Similar non-significant results were found for the bilateral heel lift task for both ipsilateral (injected side) and contralateral ankle joint: (i) peak plantar flexion angle between injections applied in the VM muscle (ipsilateral: hypertonic 108.0 ± 11.6 degrees vs isotonic 106.9 ± 10.7 degrees; contralateral: hypertonic 107.2 ± 11.3 degrees vs isotonic 107.7 ± 10.8 degrees) or TA muscle (ipsilateral: hypertonic 107.3 ± 13.4 degrees vs isotonic 110.4 ± 11.2 degrees; contralateral: hypertonic 108.0 ± 11.9 degrees vs isotonic 109.6 ± 10.3 degrees); and (ii) the peak angular velocity of the ankle joint between injections applied in the VM muscle (ipsilateral: hypertonic 168.3 ± 26.5 degrees/s vs isotonic 162.9 ± 21.4 degrees/s; contralateral: hypertonic 171.1 ± 27.3 degrees/s vs isotonic 166.7 ± 24.1 degrees/s) or TA muscle (ipsilateral: hypertonic 160.1 ± 21.4 degrees/s vs isotonic 174.5 ± 18.2 degrees/s; contralateral: hypertonic 166.6 ± 22.4 degrees/s vs isotonic 170.4 ± 20.2 degrees/s). All the details of the kinematics analyses including the other test conditions were published elsewhere [31].

**Table 1:** Mean (± SEM, N = 9) reaction time of target movement.

		Pain Condition	Baseline (ISO)	Isotonic Injection	Post-Injection (ISO)	Baseline (HYP)	Hypertonic Injection	Post-Injection (HYP)
Shoulder flexion	Reaction time of wrist (ms)	VM pain	337.0 ± 22.0	327.2 ± 21.4	324.6 ± 20.3	325.4 ± 23.9	336.4 ± 27.6	322.7 ± 31.7
		TA pain	311.0 ± 21.6	319.1 ± 21.3	299.3 ± 24.3	317.6 ± 20.6	326.3 ± 21.5	300.1 ± 23.6
Bilateral heel lift	Reaction time of bilateral heel (ms)							
	Ipsilateral side	VM pain	508.3 ± 34.8	517.2 ± 38.2	517.4 ± 30.2	527.0 ± 33.9	545.8 ± 35.3	516.9 ± 34.1
	Contralateral side		510.6 ± 37.8	528.7 ± 39.9	516.1 ± 34.7	525.3 ± 37.1	553.9 ± 38.5	519.1 ± 38.4
	Ipsilateral side	TA pain	512.0 ± 36.0	518.9 ± 38.6	529.2 ± 34.8	511.3 ± 35.3	540.4 ± 32.1	526.1 ± 38.1
	Contralateral side		526.2 ± 36.3	528.2 ± 35.6	536.7 ± 34.6	516.5 ± 35.4	555.1 ± 32.5	533.2 ± 39.6

The ipsilateral side is the side exposed to experimental pain. The reaction time is defined as the time from go-signal to onset of target movement (shoulder flexion task and bilateral heel lift).

### CoP displacement and velocity in baseline conditions

**Fig 2:**
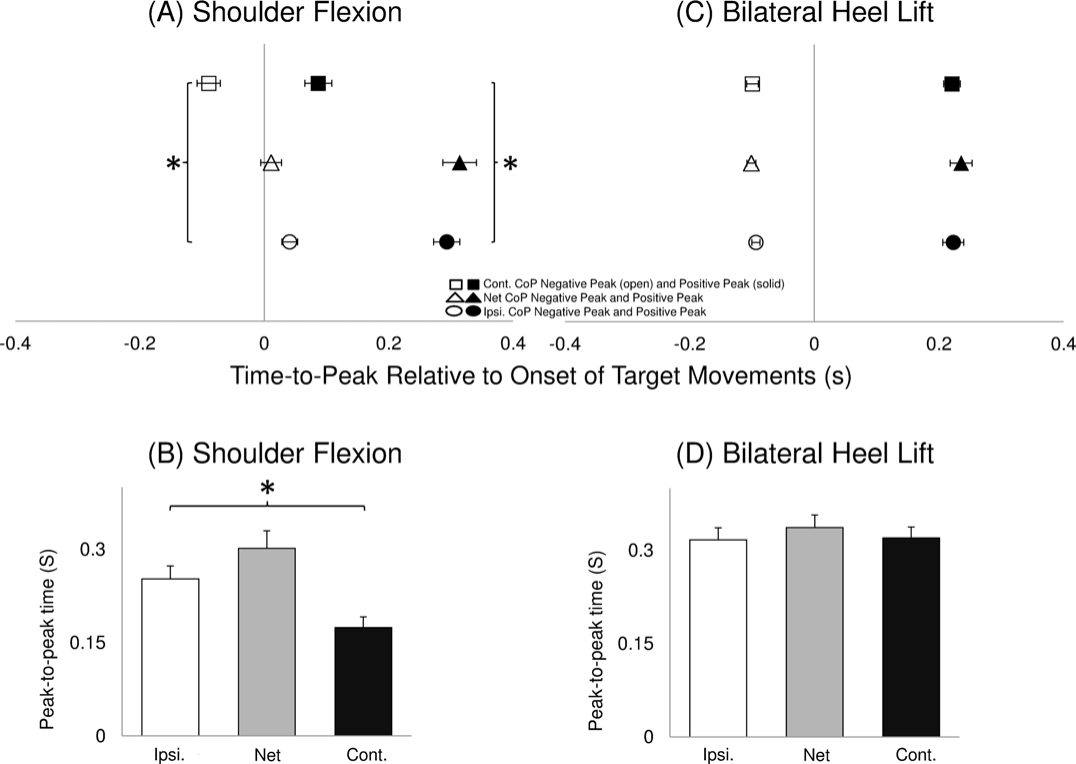
Mean (±SEM, N = 9) time for the first negative and first positive peak of AP direction across the four baseline sessions of CoP displacement during unilateral shoulder flexion (A) and bilateral heel lift (C). The time for negative (open symbols) and positive (solid symbols) peaks are illustrated for the ipsilateral (circles), net CoP (triangles) and contralateral (squares). The symbol “*” indicates significant earlier peak time of contralateral compared with the ipsilateral CoP. Time duration between two peaks of AP direction across the four baseline sessions of CoP displacement during unilateral shoulder flexion (B) and bilateral heel lift (D). Ipsilateral (open bars), net CoP (grey bars) and contralateral (solid) are illustrated. The symbol “*” indicates significantly longer time of the ipsilateral side compared with the contralateral side is illustrated.

The time-to-peak relative to the target movement onset (Fig 2A and 2C) and peak-to-peak time interval (Fig 2B and 2D) are illustrated averaged across all baselines. During the unilateral shoulder flexion task, both negative and positive peaks in the ipsilateral side were delayed in relation to the contralateral side (RM-ANOVA: F (1,8) >51.47, P < 0.01; NK: P < 0.01). Additionally, the ipsilateral peak-to-peak time interval during the unilateral shoulder flexion was longer when compared with the contralateral side (RM-ANOVA: F (1,8) > 18.46, P < 0.01; NK: P < 0.01).

**Fig 3:**
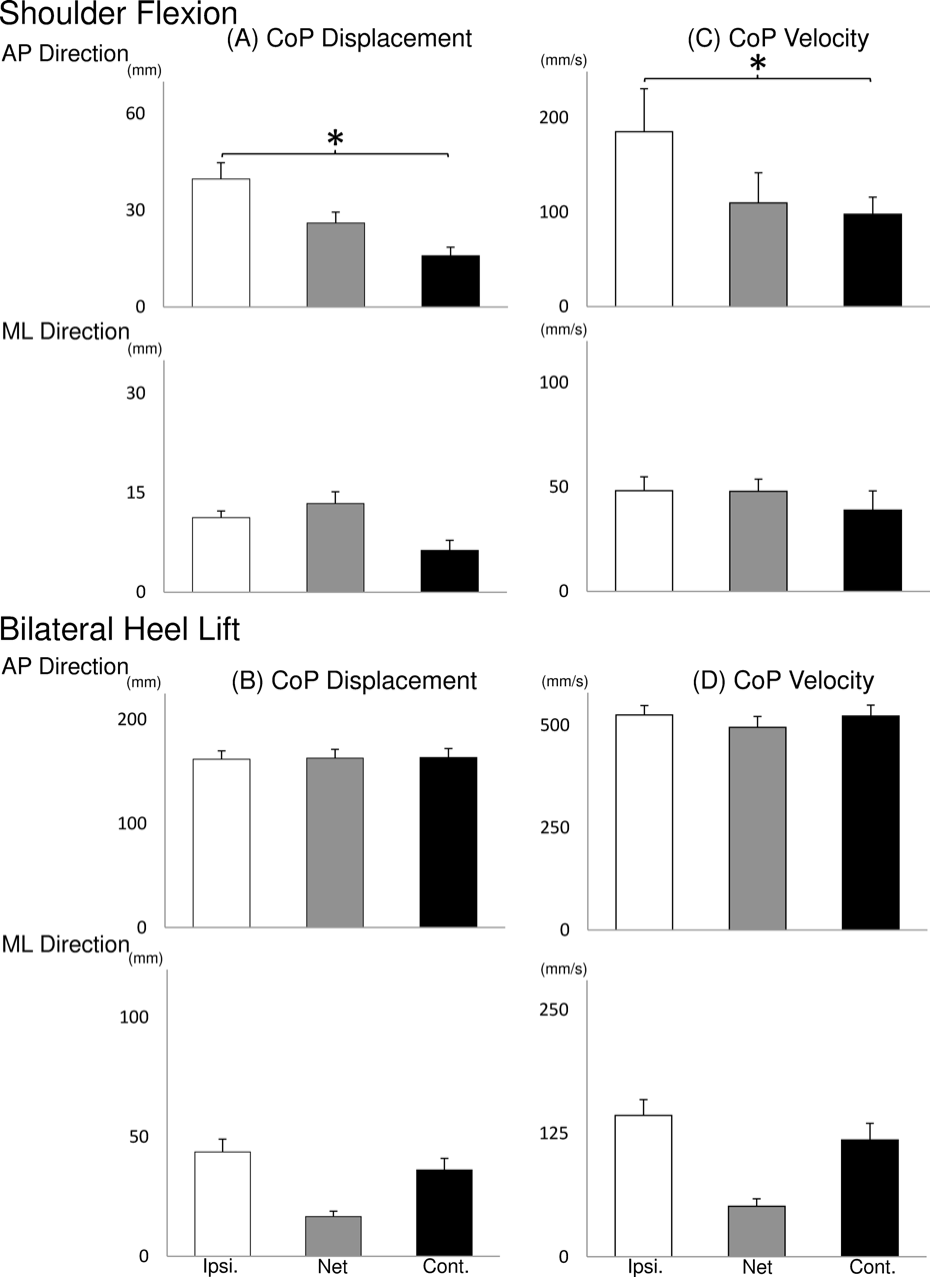
Mean (± SEM, N = 9) CoP displacement and velocity between CoP peak to peak averaged across the four baseline sessions. Ipsilateral (open bars), net CoP (grey bars) and contralateral (solid) displacements are illustrated. The symbol “*” indicates significant increase compared with the contralateral CoP.

The CoP peak-to-peak displacement (Fig 3A and 3B) and velocity (Fig 3C and 3D) for the ipsilateral, contralateral and net CoP averaged across all baselines are shown. The ipsilateral CoP peak-to-peak displacement and velocity in the AP direction during unilateral shoulder flexion task was larger than the contralateral side (RM-ANOVA: F (1,8) > 6.14, P < 0.05; NK: P < 0.05).

### CoP displacement during pain

**Fig 4:**
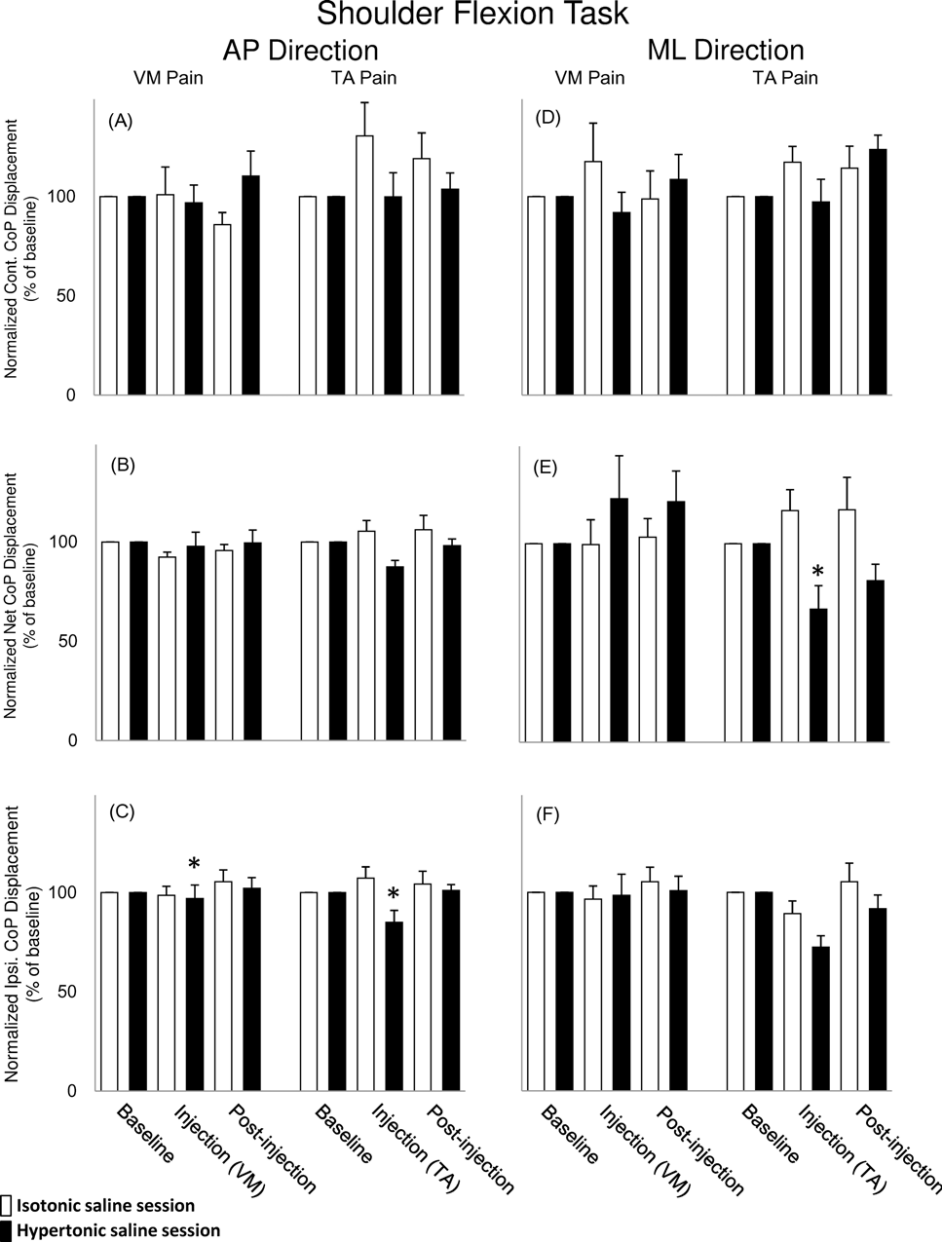
Mean (± SEM, N = 9) normalized peak to peak displacement in the anterior-posterior (AP) CoP directions (A-C) and the medial-lateral (ML) CoP directions (D-F) during unilateral shoulder flexion task. The data is illustrated for the ipsilateral (Ipsi.), the net CoP, and the contralateral (Cont.) displacement during baseline, injection, and post-injection. CoP displacement in isotonic saline series (open bar) and hypertonic saline series (solid) saline injected into the VM and TA muscles are illustrated. The symbol “*” indicates significant smaller displacement compared with non-pain condition is illustrated.

During unilateral shoulder flexion task, a significant interaction between *saline* and *time* was found for the normalised ipsilateral CoP displacement in the AP direction where it was reduced during pain when compared with the non-painful condition (Fig 4C, RM-ANOVA: F(2,16) = 5.90, P < 0.05; NK: P < 0.01). Moreover, in the unilateral shoulder flexion a significant interaction between *saline*, *time* and *painful muscle* was found for the normalised net CoP displacement in the ML direction; during the TA pain condition it was reduced when compared with the non-painful condition (Fig 4E, RM-ANOVA: F(2,16) = 3.67,P < 0.05; NK: P < 0.05).

## Discussion

This study investigated the effects of muscle pain close to the knee joint on the anticipatory postural adjustments during fast movements and the contribution of the CoP under each foot to the total body stability. The novel results show that for the fast unilateral shoulder flexion movement, the net and ipsilateral peak-to-peak CoP displacement in the ML and AP directions were reduced with pain in the TA muscle. Regardless the location, painful stimuli did not alter the movement onset, peak for angular position and velocity and time-related CoP parameters during bilateral heel lift or unilateral shoulder flexion task.

### Difference of CoP parameters between ipsilateral and contralateral side

Using two force platforms under each foot, the novel results of the present study allowed not only to quantify the independent dynamic contribution of each leg (ipsilateral and contralateral to pain) when controlling balance, but also the final neuromuscular output represented by the net CoP [33]. Previous balance studies using two force platforms showed that Parkinson’s patients produced more and longer APAs prior to step initiation induced by an external perturbation when compared with healthy participants [38,39]. In the unilateral shoulder flexion task of this study, analyses of the postural sway during the baseline conditions revealed that the ipsilateral side when compared with the contralateral side had (i) delayed time to negative and positive CoP peak, (ii) longer CoP peak-to-peak time interval, and (iii) larger CoP peak-to-peak displacement and velocity in the AP direction. Interestingly, the difference between sides (asymmetry) of CoP time-to-peak, time interval between two peaks and velocity found during the baseline condition were also maintained during the painful conditions except for the normalised displacement in the AP direction. The APA mechanisms indicated a motor strategy inherent to the task performed since this asymmetry has been preserved during painful conditions. No difference between sides in any variable analysed was found during the bilateral heel lift task regardless the presence or not of pain, therefore keeping the movement symmetry during all conditions. These results suggest that the central nervous system during a painful condition prioritise the suppressing of pain effects on the posture control strategy. Nonetheless, the ipsilateral CoP AP displacement during unilateral shoulder flexion was reduced by approximately 10%, partly reducing the asymmetry. Therefore this might be an indication that the system aims to maintain the movement patterns (asymmetry for unilateral shoulder flexion and symmetry for bilateral heel lift task) similar to the one adopted during non-painful conditions. When the injected side was the prime mover (unilateral shoulder flexion movement) smaller displacement was observed in the ipsilateral CoP, probably indicating a protective strategy to decrease load in the painful limb [40].

### Pain effects on the peak-to-peak displacement of CoP during APA

Experimental pain close to the knee joint affected gait [5], forward lunge [7], force steadiness [14] and the maximum voluntary extension of knee extension force [12,13] in healthy subjects. In previous studies, experimental knee and leg muscle pain impaired postural control in AP and ML directions during quiet standing compared with the non-painful condition [15,40]. In the present study, the central nervous system attempts to maintain the posture control strategy similar to the non-painful condition (by maintaining the asymmetrical movement pattern during the unilateral shoulder flexion task), however, the results suggests that pain altered the motor output when compared with pain free conditions. Painful stimuli (regardless if the painful location was in the TA or VM muscle) induced reduction of the normalised CoP displacement in AP direction in the painful side during unilateral shoulder flexion task, however, only pain in the TA muscle reduced the net CoP displacement in the ML direction. Recently, Karayannis et al [41] showed in low back pain patients a significant association between increased trunk stiffness and fear of movement. Although this study did not measure either joint stiffness or fear of movement, the decreased CoP displacement is an indication that the total body movement during pain was decreased, which could be related with increased stiffness in relevant postural joints. Another example, where smaller net CoP displacement during APAs was associated with suboptimal motor response, was shown in Parkinson patients out of medication and consequent smaller net CoP displacement during APA [42]. Interestingly, when these patients restart the medication, net CoP displacement increased and approached values obtained in the control group [42]. Therefore, the changes found in the net CoP in the present study were probably driven by the painful stimuli during unilateral shoulder flexion movement. Additionally, painful injection into the TA muscle induced referred pain around the ankle joint, which may have affected the motor response on the muscles located in this area [31,43]. In addition, pain in the TA muscle affected the APA mechanisms probably because the TA muscle has an important biomechanical role for controlling the ankle joint and modulation of foot pressure during standing [44]. The changes in the painful limb affected the net CoP displacement, which indicates that not only the total body balance was disturbed, but also the non-painful limb was unable compensate the loss of balance control from the painful limb.

Experimental pain has different effects on each task as observed in CoP displacement, indicating that the APA strategies are different in each of the tasks evaluated. For example, TA muscles have a great participation in the APA generation during unilateral shoulder flexion to counterbalance the perturbation caused by the fast arm movement [45], however, during heels lift tasks, the TA muscle is not only responsible for APAs but also for executing the movement itself [31].

It was surprising that VM pain did not affect the displacement of net CoP in the unilateral shoulder flexion task since previous studies reported impaired postural control due to experimental pain on the quadriceps femoris muscle [7]. One possibility is that postural muscle onset during APAs was effectively reorganised during pain in the VM [31], therefore maintaining the net CoP displacement within ranges comparable with non-painful conditions.

Pain did not affect any postural CoP parameter during bilateral heel lift task, although it has been shown previously that such paradigm affect the onset activity of different postural muscles [31]. When compared with unilateral shoulder flexion task, the reaction time for the bilateral heel lift task was, on average, 150 ms longer. This delay might provide adequate processing time for the central nervous system to establish a more accurate postural response to overcome the effects of pain (such as time reorganization in the onset of the muscles activities while performing the bilateral heel lift task [31].

These results indicate that during experimental muscle pain close to the knee joint, the central nervous system in healthy individuals is robust in maintaining the APAs motor strategy similar to what is observed in pain free conditions. Although similar experimental pain models have shown good validity in clinical settings, translation of these results to real chronic pain patients should be done with caution since muscle structures are usually not the source of pain in this population. Additionally, in chronic pain patients, other factors that were not accounted for in our experimental model also affect balance of APAs, for example as age [21,46–53], gender differences [54, 55], muscle strength [56–58] and cognition [48, 59–60]. Finally, the definition of APAs used in this study (time window: 50ms prior movement onset until 150ms after movement onset) likely includes early reflex contributions to the perturbation that would be classed as the compensatory postural adjustment (time window: +100 to +250ms from the prime mover muscle onset) [61]. Nonetheless, the present results showed that the quality of the motor response during experimental pain is suboptimal, affecting balance stability in healthy subjects when performing fast movements. The results also indicate a distinct adaptation to pain between the ipsilateral and contralateral lower limbs. Perhaps this indicates an attempt of the central nervous system to use the resources of the non-painful side in other to compensate the effects of pain in the ipsilateral side. Additionally, given that the included subjects did not have other confounding factors (e.g. structural changes in the joints), the changes observed in the motor responses also points towards a pain-driven impairments from the ipsilateral side. Rehabilitation strategies aiming to enhance muscular responses and decrease pain intensity in the painful limb may lead to APAs improvements in subjects reporting pain close to the knee joint.

## Supporting Information

S1 Table Experimental Data (Excel file). The excel archive contains 13 sheets (one for pain scores and 12 for CoP variables) with respective data for all 9 subjects (identified as “ID” column in all sheets). For the “Pain-score” sheet, pain scores are reported for both type of saline injections (isotonic and hypertonic) in the vastus-medialis (VM) muscle and tibialis-anterior (TA) muscle. Center of pressure displacement (named “DIS” in the respective sheets) and velocity (named “VEL” in the respective sheets) are reported for both directions [medial-lateral (ML) and anterior-posterior (AP)] for the ipsilateral (IPI), contralateral (CON), and net (NET) components during shoulder flexion and bilateral heel lift tasks.
(XLSX)
